# RPN1 at the crossroads of glycosylation, tumor immunity, and disulfidptosis

**DOI:** 10.3389/fphar.2026.1911035

**Published:** 2026-08-21

**Authors:** Xiaoxi Chang, Linjun Weng, Weiquan Yu, Yan Shen

**Affiliations:** 1 School of Gongli Hospital Medical Technology, University of Shanghai for Science and Technology, Shanghai, China; 2 Pudong Gongli Hospital, Shanghai University of Medicine and Health Sciences, Shanghai, China; 3 Tongji University Cancer Center, School of Medicine, Tongji University, Shanghai, China

**Keywords:** disulfidptosis, glycosylation, PDL1, Rpn1, tumor immunity

## Abstract

Ribophorin I (RPN1), a core component of the oligosaccharyltransferase complex, is traditionally known for its role in endoplasmic reticulum-associated N-glycosylation. Recent studies have identified RPN1 as an emerging regulator of tumor progression and immunity. Aberrant RPN1 overexpression has been reported in multiple malignancies, including glioma, hepatocellular carcinoma, sarcoma, and triple-negative breast cancer, where it is frequently associated with aggressive clinicopathological features and poor prognosis. RPN1 promotes tumor immune evasion by promoting N-glycosylation and stabilization of programmed death-ligand 1 (PD-L1), thereby enhancing immune checkpoint signaling and directly inhibiting anti-tumor T-cell responses. Consequently, elevated RPN1 expression is consistently associated with an immunosuppressive tumor microenvironment rich in M2 macrophages and poor in CD8^+^ T cells. More importantly, multiple omics signature analyses indicate RPN1 is integrated into several disulfidptosis-related risk models; however, direct experimental evidence confirming the causal linkage between RPN1 and disulfidptosis remains limited. Correlative database data also show potential associations between RPN1 upregulation and genomic instability and treatment resistance. Based on tiered classification of existing evidence (biochemical functional validation vs. multi-omics correlation), this review systematically summarizes the biological roles of RPN1 in cancer, its functions in tumor immunity and disulfidptosis-associated pathways and finally evaluates its potential as a therapeutic target in precision oncology.

## 1 Introduction

### 1.1 Basic biological functions of RPN1

**FIGURE 1:**
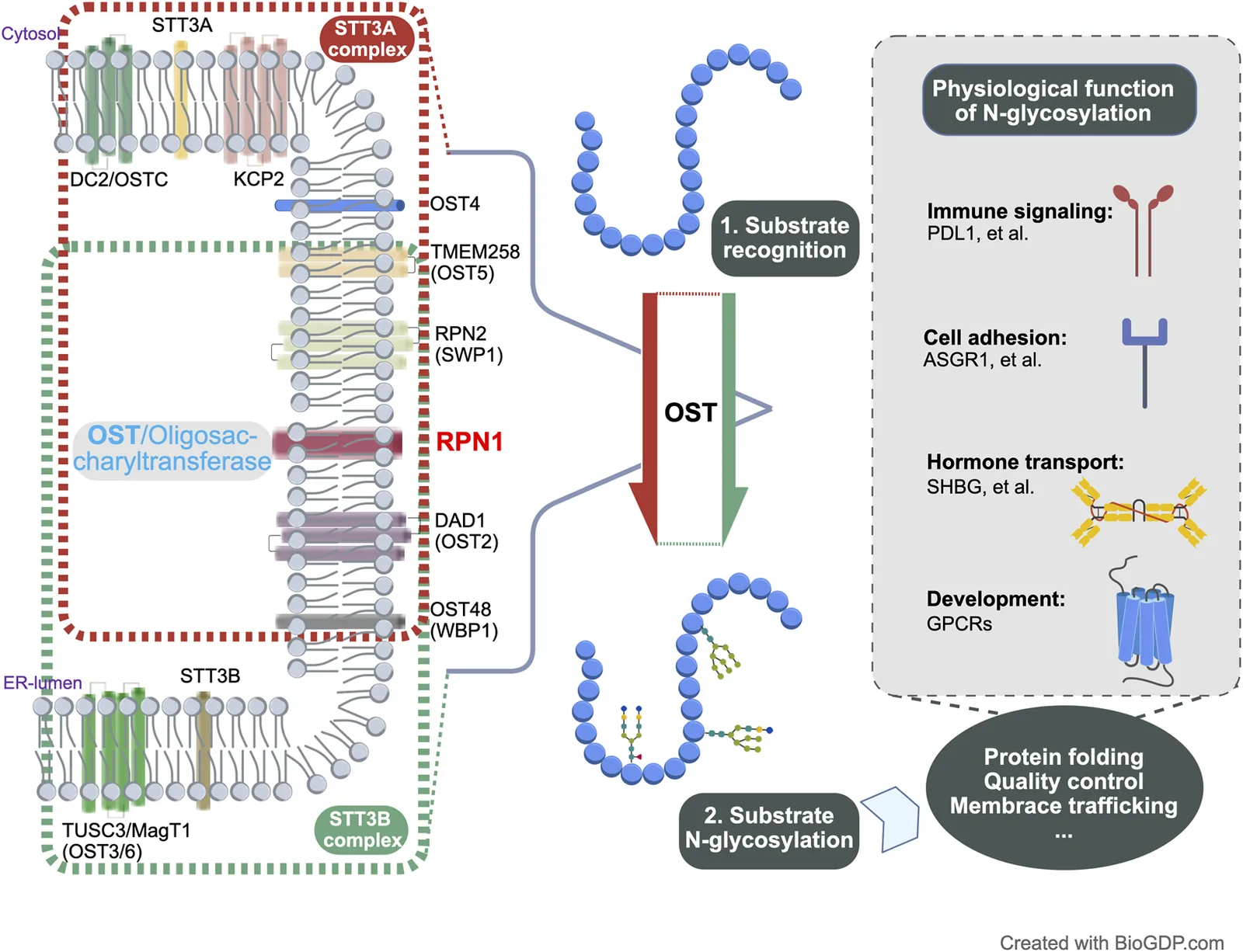
Structure and biological functions of RPN1 in the oligosaccharyltransferase (OST) complex. The OST complex is an ER-resident multi-subunit enzyme that catalyzes co-translational N-glycosylation of nascent polypeptides. The complex comprises a catalytic core (STT3A or STT3B) and accessory subunits including RPN1, RPN2, DAD1, and others. As a core component, RPN1 functions in substrate recognition and is required for efficient N-glycosylation, which is critical for subsequent protein folding, quality control, and membrane trafficking. Representative substrates and associated physiological processes include immune signaling (PD-L1), cell adhesion (ASGR1), hormone transport (SHBG), and developmental regulation (GPCRs). OST, oligosaccharyltransferase; ER, endoplasmic reticulum; N-glycosylation, N-linked glycosylation.

RPN1 (Ribophorin I) is an essential subunit of the oligosaccharyltransferase (OST) complex in the endoplasmic reticulum (ER), where it plays a central role in N-glycosylation (Elsasser et al., 2002) (Figure 1). This highly conserved co-translational modification transfers pre-assembled high-mannose oligosaccharides from a lipid carrier to specific asparagine residues on nascent polypeptides, a step critical for protein folding, quality control, sorting, and functional maturation (Helenius and Aebi, 2004). Within the multi-subunit OST complex, STT3 provides the catalytic core, while RPN1 functions as a non-catalytic but indispensable auxiliary subunit that handles substrate recognition and delivery (Wilson et al., 2008).

RPN1 binds nascent glycoprotein precursors through its ER lumenal domain and presents them to STT3, facilitating efficient, site-specific N-glycosylation (Wilson et al., 2008). Although not absolutely required for the reaction, RPN1 markedly enhances glycosylation efficiency for select membrane and secretory proteins, pointing to a substrate-selective regulatory role (Wilson and High, 2007). Knocking down RPN1 by RNA interference lowers N-glycosylation of certain membrane proteins—the mu1 opioid receptor being one example—impairing their folding and transport to the cell membrane (Cho et al., 2024). These observations suggest a chaperone-like function that guides substrates into the OST catalytic center (Wilson and High, 2007).

RPN1 has a role beyond catalysis: it acts as a structural scaffold that is essential for maintaining OST complex stability, as elegantly demonstrated by the DAD1 temperature-sensitive mutant line tsBN7, in which DAD1 degradation leads to simultaneous loss of RPN1 and other OST subunits and hence to a complete loss of global N-glycosylation capacity (Sanjay et al., 1998). Therefore, the subunits are tightly interdependent, and RPN1 expression directly affects the functional integrity of the entire complex.

RPN1 may also modulate downstream glycan processing through interactions with other enzymes. It binds the Golgi-resident core fucosyltransferase FUT8 via an SH3 domain-dependent mechanism, enhancing FUT8 activity and thereby affecting core fucose modification levels (Tomida et al., 2020). Although this interaction occurs in the Golgi, it indicates RPN1 can exert regulatory influence at multiple nodes along the N-glycosylation pathway, coupling initial ER glycosylation to subsequent glycan maturation.

Under physiological conditions, RPN1-mediated N-glycosylation is fundamental to immune receptor signaling, hormone transport, cell adhesion, and neural development (Trombetta, 2003; Helenius and Aebi, 2004; De Haas et al., 2020; Qin et al., 2024; Zhang M. et al., 2025). Moreover, two important physiological proteins, sex hormone-binding globulin and the asialoglycoprotein receptor 1, absolutely require efficient RPN1-mediated glycosylation for proper cell surface expression (Cho et al., 2024). Therefore, RPN1 functions not only as a regulator of the N-glycosylation machinery but also as a molecular hub that coordinates protein homeostasis and normal cellular function. This central role in protein fate naturally leads to the critical question: what are the consequences of RPN1 aberrations in pathological states, especially in tumorigenesis? (Wilson and High, 2007; Wilson et al., 2008; Guay et al., 2025).

### 1.2 Links between RPN1 and tumor development

Because RPN1 plays such a central role in protein homeostasis, aberrant RPN1 expression has become a subject of growing interest in cancer research. Transcriptome, proteome and retrospective immunohistochemical cohort analyses from multiple independent groups consistently report elevated RPN1 expression in triple-negative breast cancer (TNBC), glioblastoma (GBM), hepatocellular carcinoma (HCC), sarcoma, and non-muscle-invasive bladder cancer (NMIBC) (Wang et al., 2023b; Zong et al., 2024; Wang M. et al., 2025; Zhang R. et al., 2025). More importantly, elevated RPN1 expression is consistently linked to aggressive clinicopathological features and poor clinical outcomes.

In TNBC, both mRNA and protein levels of RPN1 are significantly increased compared with normal breast tissues. Functional studies have shown that RPN1 knockdown suppresses cell proliferation, migration, invasion, and colony formation, whereas ectopic expression promotes these malignant phenotypes (Wang M. et al., 2025). Similar observations have been reported in HCC, where RPN1 expression is significantly higher in tumor tissues than in adjacent non-tumor tissues. Interestingly, RPN1 expression varies across different stages of HBV-associated liver disease, suggesting that it may participate in early molecular events during hepatocarcinogenesis (Zhang R. et al., 2025). In NMIBC, immunohistochemical analyses have demonstrated a positive association between RPN1 expression and tumor size, pathological stage, histological grade, and postoperative recurrence (2023).

**FIGURE 2:**
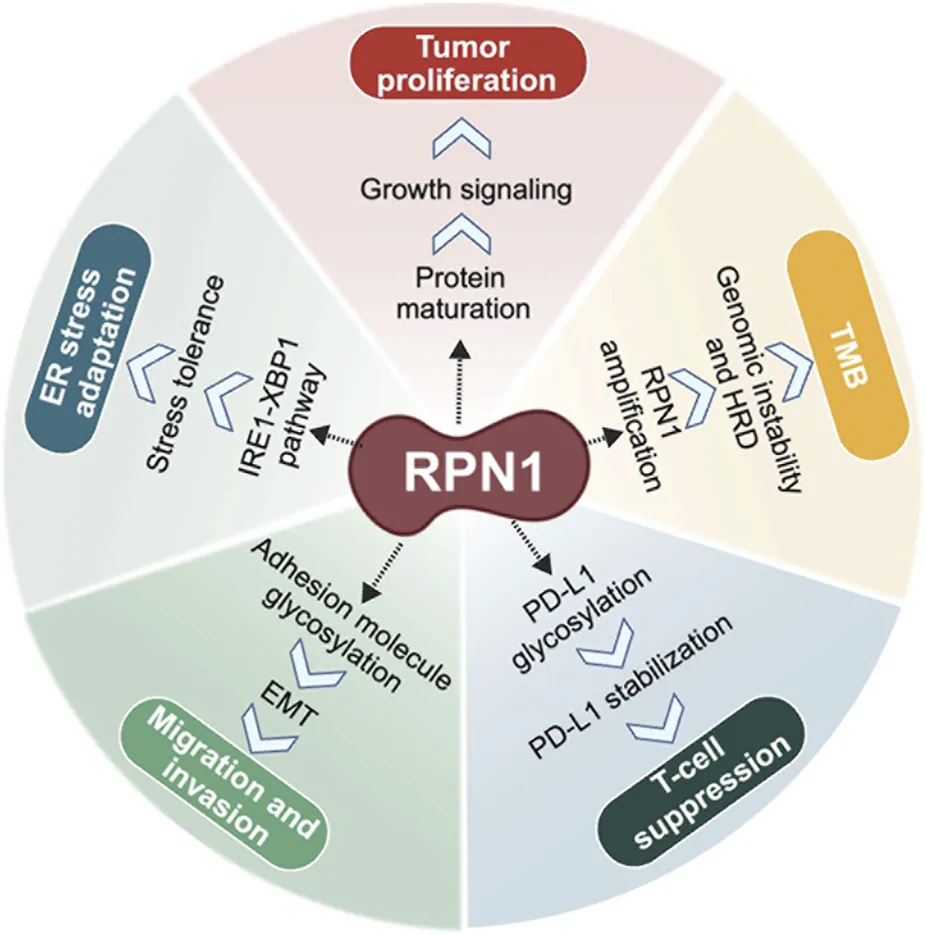
Multifaceted oncogenic roles of RPN1 in tumor progression. As a core subunit of the oligosaccharyltransferase (OST) complex, RPN1 drives malignant progression through five key biological axes: (1) Tumor proliferation: by supporting the glycosylation and maturation of growth signaling proteins, RPN1 sustains proliferative signaling pathways; (2) Migration and invasion: it mediates the glycosylation of adhesion molecules, promoting epithelial–mesenchymal transition (EMT), cytoskeleton remodeling, and metastatic dissemination; (3) ER stress adaptation: via the IRE1–XBP1 pathway, RPN1 enhances stress tolerance, enabling tumor cell survival under harsh microenvironmental conditions; (4) Genomic instability: RPN1 amplification correlates with homologous recombination deficiency (HRD), driving elevated tumor mutational burden (TMB) and tumor evolution; (5) Immune evasion: RPN1-mediated PD-L1 glycosylation stabilizes the immune checkpoint protein, leading to enhanced T-cell suppression. HRD, homologous recombination deficiency; TMB, tumor mutational burden; EMT, epithelial–mesenchymal transition.

The oncogenic role of RPN1 goes well beyond its effects on tumor cell proliferation, since RPN1 is a fundamental component of the N-glycosylation machinery and therefore directly affects the maturation, stability, and trafficking of a large number of membrane and secreted proteins. In breast cancer models, RPN1 has been shown to promote PD- L1 glycosylation and enhance PD-L1 protein stability, which in turn facilitates immune evasion (Wang M. et al., 2025). Thus, there is strong evidence that RPN1 contributes both to intrinsic tumor cell fitness and to the regulation of tumor–immune interactions (see Figure 2).

Clinical studies have provided correlative evidence for the prognostic value of RPN1: in a series of 196 breast cancer patients, high RPN1 protein expression was directly associated with lymph node metastasis, vascular invasion, shorter relapse-free survival, and worse overall survival (Milde-Langosch et al., 2015). Pan-cancer omics mining using TCGA and GTEx datasets further indicated RPN1 upregulation across tumor types, and its expression correlates with genomic instability-related features, including tumor mutational burden (TMB) (Zheng and Song, 2024).

Collectively, existing stratified evidence (cohort correlation + partial cell/mouse functional validation) implies RPN1 overexpression participates in multi-step tumor progression and predicts unfavorable prognosis and immune resistance, which provides rational grounds to systematically summarize its tumor-related biological functions and translational potential.

### 1.3 Literature search strategy

To construct this narrative review with minimized literature selection bias, standardized retrieval and two-step screening workflows were implemented. Literature retrieval was conducted up to June 2026 across four core databases: PubMed, Web of Science Core Collection, Embase, and CNKI. Mesh terms and free-test keywords covering RPN1, OST complex, N-glycosylation, PD-L1, tumor immune escape, disulfidptosis, and solid malignancies were combined with Boolean operators AND/OR were to expand retrieval coverage.

Inclusion criteria: peer-reviewed original articles and formal reviews focusing on RPN1’s glycosylation function, tumor regulatory roles, prognostic signatures and therapeutic potential; studies containing *in vitro* biochemical assays, genetically modified cell models, xenograft/syngeneic mouse experiments, or large-scale TCGA/CPTAC clinical omics cohorts.

Exclusion criteria: non-tumor basic research in yeast/plants; conference abstracts, preprint manuscripts, case reports without systematic mechanism elaboration; duplicate datasets published by identical research teams; pure omics correlation papers without further functional validation.

All retrieved records were de-duplicated, followed by title/abstract screening by twe independent authors. Full-text reading was conducted for secondary inclusion evaluation, and disagreements over inclusion standards were resolved via discussion with the corresponding author.

Unlike previous reviews focusing on pan-cancer RPN1 expression or disulfidptosis separately, this review positions RPN1 at the crossroads of glycosylation, tumor immunity, and disulfidptosis. We integrate current evidence linking RPN1-dependent N-glycosylation with PD-L1 stabilization, immune evasion, and disulfidptosis-associated pathways, while distinguishing established mechanisms from emerging hypotheses. We further discuss the translational potential and current challenges of targeting RPN1, including safety, delivery, and its context-dependent value as a biomarker for immunotherapy.

## 2 RPN1 expression and prognostic value across cancers

### 2.1 Differential expression in multiple cancers

Multi-omics mining based on TCGA, GTEx and CPTAC public datasets reveals a widespread trend of RPN1 transcriptional and protein overexpression across 33 tumor types, with the highest expression detected in highly malignant subtypes including glioma, HCC, sarcoma and TNBC; this conclusion is limited to database correlation without unified pan-cancer animal verification (Luo et al., 2025).

In gliomas, RPN1 mRNA and protein both exceed levels in normal brain tissue, and expression closely tracks pathological grade in high-grade gliomas including GBM (Guay et al., 2025). In HCC, RPN1 is significantly elevated in tumor versus adjacent normal tissue (p < 0.001), and its expression in peripheral blood mononuclear cells (PBMCs) also surpasses that in healthy controls, hinting at liquid biopsy utility (Zhang R. et al., 2025). Notably, RPN1 expression is higher during the HBV-related cirrhosis stage than in the HCC stage, suggesting involvement in early hepatocarcinogenesis (Zhang R. et al., 2025).

Sarcomas exhibit consistent RPN1 overexpression, and elevated mRNA levels are correlated with adverse outcomes (Luo et al., 2025). More importantly, in TNBC, a refractory subtype that lacks hormone receptors and HER2 amplification, RPN1 overexpression has been repeatedly documented. Wang et al. showed that high RPN1 levels are present in TNBC cell lines and clinical specimens by RT-qPCR and immunohistochemistry, and they directly linked high RPN1 to increased proliferation, migration, and decreased survival (Wang M. et al., 2025). Earlier protein-level analysis of 196 breast cancer samples had already established that RPN1 overexpression correlates with vascular invasion, lymph node metastasis, and shorter relapse-free and overall survival (Milde-Langosch et al., 2015).

Cross-cancer omics comparisons indicated RPN1 upregulation is a general, pan-cancer phenomenon rather than restricted to a particular tissue origin. Luo et al. analyzed 33 cancers using TCGA and GTEx data and found RPN1 transcript abundance markedly higher in tumor tissues than matched normal counterparts (Feng et al., 2024; Luo et al., 2025). CPTAC proteomic data further supported this transcriptional trend across solid tumors (Luo et al., 2025).

Combining transcriptome and proteome correlative data, we infer RPN1 upregulation participates in universal tumorigenesis-related pathways and holds potential as prognostic and therapeutic target marker.

### 2.2 Association with clinicopathological features and prognosis

Retrospective clinical cohort analyses indicated elevated RPN1 expression correlates with high pathological grade, advanced TNM stage, lymphatic metastasis and inferior survival across multiple tumors, especially in GBM. A large multi-cohort study of 1,592 glioma patients showed that RPN1 expression increases during progression from low-grade glioma to GBM and is strongly associated with unfavorable molecular markers: IDH wild-type status and unmethylated MGMT promoter (Zong et al., 2024). When integrated into disulfidptosis risk scoring systems, high RPN1 expression corresponds to inferior survival, and multivariate Cox regression identified RPN1 as an independent prognostic variable (Wang et al., 2023a).

In HCC, high RPN1 expression correlates with poor tumor differentiation and shortened overall survival, which supports its potential value as an independent prognostic biomarker (Zheng et al., 2022).

Pan-cancer omics extended this correlative observation to sarcoma and other solid tumors; across 33 cancer datasets, high RPN1 transcript abundance correlates with advanced TNM stage and distant metastasis (Luo et al., 2025). More importantly, in sarcoma, high RPN1 levels independently predict shortened progression-free and overall survival even after proper adjustment for age, sex, and stage in multivariate Cox regression (Luo et al., 2025).

Collective correlative clinical data show high RPN1 expression is tightly linked to malignant clinicopathological phenotypes and inferior prognosis in GBM, HCC, sarcoma and TNBC, and multivariate statistical models support its independent prognostic capacity in multiple cohorts, implying RPN1 is a candidate tumor-promoting factor with clinical predictive value.

### 2.3 Genomic variations and genomic instability

Beyond transcriptional and protein-level overexpression, pan-cancer sequencing datasets revealed frequent RPN1 copy-number amplification, a typical molecular signature of genomic instability. Omics correlation analyses indicated RPN1 amplification positively correlates with its mRNA and protein abundance, suggesting a gene dosage effect that may facilitate tumor progression (Lin et al., 2025). Furthermore, RPN1 amplification correlates with HRD, the state of defective double-strand break repair resulting from loss of function in critical DNA repair genes, a mechanism that naturally promotes chromosomal abnormalities and genomic instability (Zhao et al., 2018). Statistical analysis by Lin et al. linked RPN1 amplification with elevated HRD scores in sarcoma and glioma, and hypothesized RPN1 overexpression indirectly disrupts DNA repair signaling to aggravate genome chaos (Lin et al., 2025).

RPN1 genomic alterations also associate with elevated TMB. Since HRD status itself drives TMB increase—as shown in multiple independent cohorts (Li et al., 2018; Jiang et al., 2020)—the HRD state linked to RPN1 amplification likely serves as an upstream mechanism for TMB elevation. Indeed, RPN1-high or amplified samples exhibit significantly higher TMB, and pathway enrichment further confirmed co-regulation between RPN1 and multiple DNA repair cascades (Lin et al., 2025).

HRD exerts divergent immune effects across distinct tumor types (Li et al., 2018). In most cancers, HRD correlates with a “cold” tumor phenotype, characterized by reduced T cell infiltration and diminished immuneactivation signals, which is partially attributed to chromosomal instability-driven release of immunosuppressive factors (Budczies et al., 2022). Therefore, RPN1, which promotes HRD and TMB elevation, can in certain contexts create a high-mutation-burden yet immunosuppressive microenvironment. This elegant paradox helps clarify why high RPN1 predicts ICI resistance in some cancers (GBM, esophageal cancer) but correlates with sensitivity in others (bladder cancer) (Lin et al., 2025). Therefore, RPN1 genomic alteration is a common genomic aberration that correlates with genome instability and shapes tumor immune recognition and therapeutic response. Discussion on spatial multi-omics biomarker validation can be further supported by spatial metabolomics research decoding tissue molecular spatial distribution patterns (Zhu J. et al., 2025).

To facilitate comparison across different tumor types, the current evidence supporting the oncogenic and immunological roles of RPN1 is summarized in Supplementary Table 1. The table integrates available evidence regarding cancer type, experimental model, detection methods, biological functions, prognostic significance, immune associations, and functional validation status.

## 3 RPN1 in immune microenvironment regulation

### 3.1 Formation of an immunosuppressive microenvironment

Cumulative correlative omics and partial cellular co-culture data support RPN1’s involvement in constructing immunosuppressive tumor microenvironments in glioma, HCC and sarcoma (Lin et al., 2025).

One of the most consistent observations is the positive correlation between RPN1 expression and markers of M2-like tumor-associated macrophages (TAMs), such as CD163 and CD206 (Lin et al., 2025). In glioma, multiplex immunofluorescence analyses have shown that regions with high RPN1 expression frequently exhibit increased infiltration of M2-polarized macrophages or microglia (Duffuler et al., 2024; Liao et al., 2025). Furthermore, co-culture experiments demonstrated that RPN1 knockdown reduced the ability of glioma cells to promote M2 polarization, as reflected by decreased expression of Arg1 and IL-10 (Lin et al., 2025). Although the underlying mechanisms remain unclear, altered secretion of glycosylated cytokines, chemokines, or extracellular vesicle-associated factors may contribute to this phenomenon.

Elevated RPN1 expression consistently correlates with decreased intratumoral cytotoxic CD8^+^ T cell infiltration across multiple tumor subtypes (Lin et al., 2025), a phenomenon that has now been well documented in multiple tumor types and is very plausibly explained by the immunosuppressive environment created by M2 macrophages (Li J. et al., 2025; Zhu, 2025; Jia et al., 2026). These cells secrete inhibitory cytokines such as IL-10 and TGF-β, both of which inhibit T-cell activation and favor immune tolerance. Moreover, RPN1 itself modulates immune checkpoint molecules in a way that directly limits anti-tumor T-cell responses (Qin et al., 2024; Liu et al., 2025b).

Clinically, correlative cohort data link high RPN1 expression with inferior ICI response in glioma and esophageal cancer, though direct causal experimental proof is still lacking (Lin et al., 2025). Collectively, existing data imply RPN1 contributes to immune-excluded or immune-suppressed tumor phenotypes, while detailed molecular cascades bridging RPN1 and immune cell recruitment/polarization require further functional verification.

### 3.2 RPN1-mediated PD-L1 glycosylation and immune evasion

**FIGURE 3:**
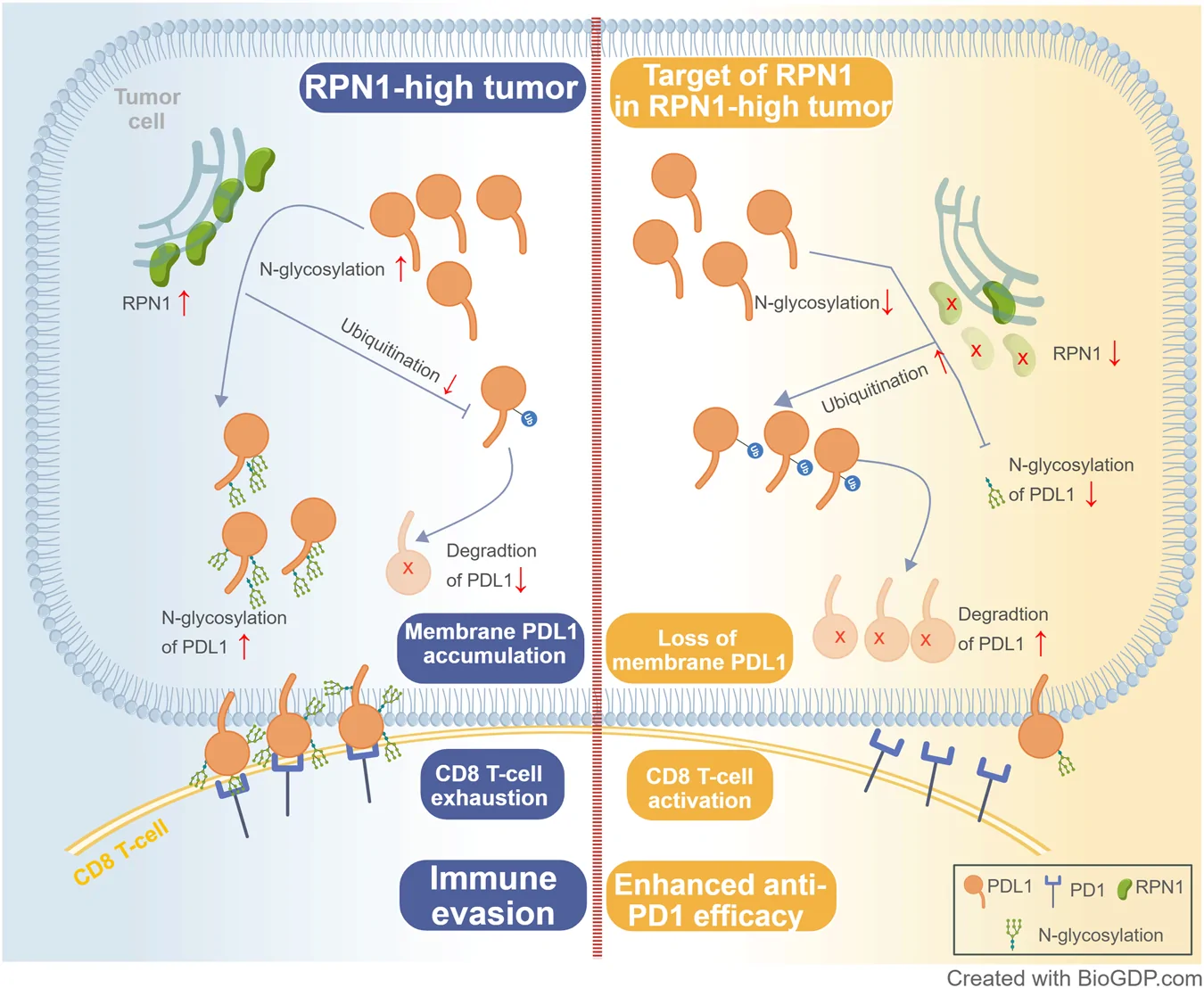
RPN1-mediated PD-L1 glycosylation and tumor immune evasion. On the left (RPN1-high tumor cells): Elevated RPN1 expression enhances the OST complex-dependent N-glycosylation of PD-L1 at key residues (N35/N192/N200/N219). This modification reduces PD-L1 ubiquitination and subsequent proteasomal degradation, leading to PD-L1 stabilization and accumulation on the tumor cell membrane. Sustained PD-L1 expression engages PD-1 on CD8^+^ T cells, promoting T-cell exhaustion, an immunosuppressive tumor microenvironment (characterized by increased M2 macrophages and Tregs, reduced CD8^+^ T cells, and impaired IFN-γ production), and ultimately immune evasion. On the right (Targeting RPN1 in RPN1-high tumors): RPN1 inhibition impairs PD-L1 N-glycosylation, increasing its ubiquitination and degradation. Reduced membrane PD-L1 expression reverses CD8^+^ T-cell suppression, restoring anti-tumor immunity and enhancing the efficacy of anti-PD-1 immunotherapy. OST, oligosaccharyltransferase; IFN-γ, interferon-gamma.

Since RPN1 is a core OST subunit, it is essential for N-linked glycosylation (Wilson et al., 2008). More importantly, multiple *in vitro* and *in vivo* biochemical experiments confirmed RPN1 enhances PD-L1 N-glycosylation, stabilizes PD-L1 protein and promotes its membrane localization to facilitate tumor immune evasion (Hwang et al., 2025) (Figure 3). Therefore, this mechanism is highly relevant in immunogenic tumors such as TNBC.

PD-L1 function is tightly controlled by post-translational modifications, especially N-glycosylation. PD-L1 contains four conserved N-glycosylation sites (N35, N192, N200, N219) whose modification maintains conformational stability, modulates PD-1 binding, and influences ICI sensitivity (Kaufman et al., 2025). RPN1 participates in oligosaccharide transfer to nascent PD-L1 asparagine residues, representing a rate-limiting step for initial PD-L1 glycosylation. In TNBC cell models, RPN1 overexpression substantially promotes PD-L1 N-glycosylation, inhibiting ubiquitination-dependent degradation, extending half-life, and enhancing membrane accumulation (Wang M. et al., 2025). RPN1 knockout decreases PD-L1 protein without altering mRNA levels, confirming a post-translational mechanism (Wang M. et al., 2025).

RPN1-mediated PD-L1 glycosylation stabilizes membrane checkpoint protein and amplifies immunosuppressive capacity. In TNBC mouse models, RPN1 deletion weakens PD-L1/PD-1 binding, restores CD8^+^ T cell cytotoxicity and suppresses *in vivo* tumor growth (Wang M. et al., 2025). More importantly, in therapy-induced senescence, chemoradiotherapy induces senescent tumor cells to upregulate RPN1, which increases PD-L1 glycosylation to form a protective “PD-L1 umbrella” that resists T cell-mediated clearance and promotes recurrence. RPN1 knockout reverses this phenotype and elevates CTL-mediated killing of senescent tumor cells (Lee et al., 2023).

PD-L1 glycosylation status determines its subcellular distribution and soluble fragment release (Kaufman et al., 2025). As an upstream regulator of initial glycan attachment, RPN1 shapes subsequent PD-L1 processing and functional output. In TNBC, RPN1 forms a well-defined regulatory axis with YY1 and YBX1 that coordinately upregulates PD-L1 expression and promotes its glycosylation, thereby elegantly integrating transcriptional and post-translational control into an immune evasion network (Wang M. et al., 2025).

Collectively, multiple independent cell and animal functional experiments confirm RPN1-dependent N-glycosylation is a key post-translational mechanism maintaining PD-L1 membrane stability and immunosuppressive activity. In TNBC preclinical models, RPN1 depletion synergizes with anti-PD-1 blockade to remodel cold tumors into immune-active phenotypes, providing preclinical evidence for glycosylation-targeted combination immunotherapy.

### 3.3 Transcriptional and signaling networks involving RPN1

Among identified upstream regulatory cascades controlling RPN1 expression, the YY1/RPN1/YBX1 axis has obtained relatively complete functional validation in TNBC. YY1 directly binds to the promoter of RPN1 and increases its transcription, leading to stabilization of YBX1, which then promotes PD-L1 expression at both transcriptional and post-transcriptional levels. Therefore, a regulatory circuit is formed that directly enhances immune evasion and tumor progression (Wang M. et al., 2025). And RPN1-mediated glycan modification complements transcriptional upregulation to maximize PD-L1 membrane abundance (Lee et al., 2023).

Beyond immune checkpoint control, RPN1 has been linked to endoplasmic reticulum (ER) stress UPR signaling, particularly the IRE1–XBP1 pathway. Loss of RPN1 impairs IRE1-mediated XBP1 splicing and thus disrupts ER stress adaptation (Larburu et al., 2024). Importantly, in esophageal squamous cell carcinoma, stabilization of RPN1 by CERS6 promotes activation of the IRE1–XBP1 pathway, which in turn enhances tumor cell survival under stressful conditions (Chen et al., 2023; Chen W. et al., 2025).

Pan-cancer omics analyses found positive correlation between RPN1 expression, HRD signature and TMB level; however, direct functional experiments confirming RPN1’s regulatory role in DNA repair pathways remain absent (Zhang R. et al., 2025). Thus, it is plausible that alterations in ER homeostasis, oxidative stress, or protein quality-control pathways affect genomic stability indirectly, but these possibilities must be tested experimentally.

Current literature supports RPN1 participates in multiple interconnected signaling networks regulating tumor immunity, ER stress adaptation and genome maintenance; systematic multi-layer mechanistic studies across distinct tumor subtypes are required to clarify their cross-talk relationships.

## 4 RPN1 and disulfidptosis

### 4.1 Disulfidptosis mechanisms and significance in cancer

Disulfidptosis is a newly described programmed cell death form that occurs as a result of disulfide stress under glucose deprivation in cells expressing high levels of SLC7A11 (Liu et al., 2024). Under physiological conditions, SLC7A11 imports extracellular cystine to support glutathione synthesis and thus maintain redox homeostasis (Liu et al., 2024; Li Y. et al., 2025). Many cancer cells overexpress SLC7A11 to enhance their antioxidant capacity, but when glucose is limited and NADPH regeneration fails, imported cystine cannot be efficiently reduced to cysteine. As a consequence, cystine accumulates and inappropriate disulfide bonds are formed (Liu et al., 2023; Mi et al., 2024; Liu et al., 2025a).

Abnormal disulfide bonds specifically target actin cytoskeleton components, triggering irreversible F-actin network contraction and cell detachment death (Liu et al., 2023; Li et al., 2024; Li Y. et al., 2025). This death pathway is biochemically and morphologically distinct from apoptosis, necroptosis, ferroptosis and cuproptosis (Jiang et al., 2021; Ma et al., 2023; Nössing and Ryan, 2023; Gu et al., 2025). The Rac1-WAVE regulatory complex pathway is directly involved, with sustained Rac1 activation accelerating disulfide-mediated cytoskeletal collapse (Liu et al., 2023; Chen et al., 2024).

Disulfidptosis reveals an exploitable metabolic vulnerability of tumors (Liu et al., 2020). Solid tumors such as GBM, HCC, and TNBC frequently overexpress SLC7A11 to cope with oxidative stress, therefore they are naturally more sensitive than normal cells to glucose limitation in nutrient-poor tumor cores or after anti-angiogenic therapy (Liu et al., 2025a). More importantly, this creates tumor-selective killing potential: normal cells with low SLC7A11 resist triggering this death pathway under glucose deficiency, whereas SLC7A11-high cancer cells become ideal targets (Li et al., 2024; Mi et al., 2024). Consequently, glucose transporter inhibitors can be used to artificially induce glucose deprivation, thereby activating disulfidptosis and effectively suppressing SLC7A11-positive tumor growth (Liu et al., 2023).

Disulfidptosis also generates dual anti-tumor benefits: cytoskeletal collapse not only kills cancer cells directly but also leads to the release of damage-associated molecular patterns (DAMPs) that promote dendritic cell maturation and T cell activation, hence enhancing anti-tumor immunity (Li et al., 2024; Mi et al., 2024; Li Y. et al., 2025). Therefore, inducing disulfidptosis represents a dual strategy with both direct cytotoxicity and the potential to synergize with ICIs to overcome resistance (Li et al., 2024; Mi et al., 2024). More broadly, as a death modality driven by metabolism–redox–cytoskeleton coupling, disulfidptosis enriches our understanding of programmed cell death and naturally points toward precision therapeutic approaches targeting tumor metabolic vulnerabilities.

### 4.2 RPN1 as a putative regulator within disulfidptosis-associated networks

Multiple pan-cancer omics signature analyses consistently identified RPN1 as a disulfidptosis-related gene sets in several cancer types (Wang et al., 2023a; Zheng and Song, 2024). Critically, unlike canonical disulfidptosis mediator SLC7A11, there exists no direct genetic or pharmacological experimental evidence proving RPN1 actively initiates or executes disulfidptosis; all existing links are limited to correlative omics enrichment data (Liu J. et al., 2025).

RPN1 is a fundamental component of the oligosaccharyltransferase complex and therefore is absolutely required for protein N-glycosylation, and it also regulates protein folding, stability, and trafficking, which in turn affect cellular responses to metabolic and oxidative stress. Since redox homeostasis is a central determinant of disulfideptosis sensitivity, it is logical to speculate that alterations in RPN1 activity could influence susceptibility to this form of cell death. However, this connection has not yet been rigorously tested experimentally (Liu et al., 2023; Qin et al., 2024).

Multi-omics enrichment and protein interaction datasets point indirectly to a possible connection between RPN1 and disulfidptosis-related pathways: RPN1 expression is already known to correlate with tumor stemness, RNA modification patterns, and cytoskeleton-related signaling networks (Zheng and Song, 2024). More importantly, protein interaction and pathway enrichment analyses have shown that RPN1-containing gene signatures are enriched in pathways regulating the actin cytoskeleton, including the RHO GTPase–WASP/WAVE signaling cascade (Zheng and Song, 2024). Since actin cytoskeleton collapse is a hallmark of disulfidptosis, these findings are highly suggestive and merit direct experimental validation.

Since RPN1 is known to be involved in tumor cell migration and invasion, which both require extensive cytoskeletal remodeling (Niu et al., 2025), it is reasonable to ask whether RPN1 directly regulates proteins participating in disulfide stress responses or actin network stability. Plausible targets would be membrane receptors, adhesion molecules, or signaling proteins that depend on correct glycosylation.

**FIGURE 4:**
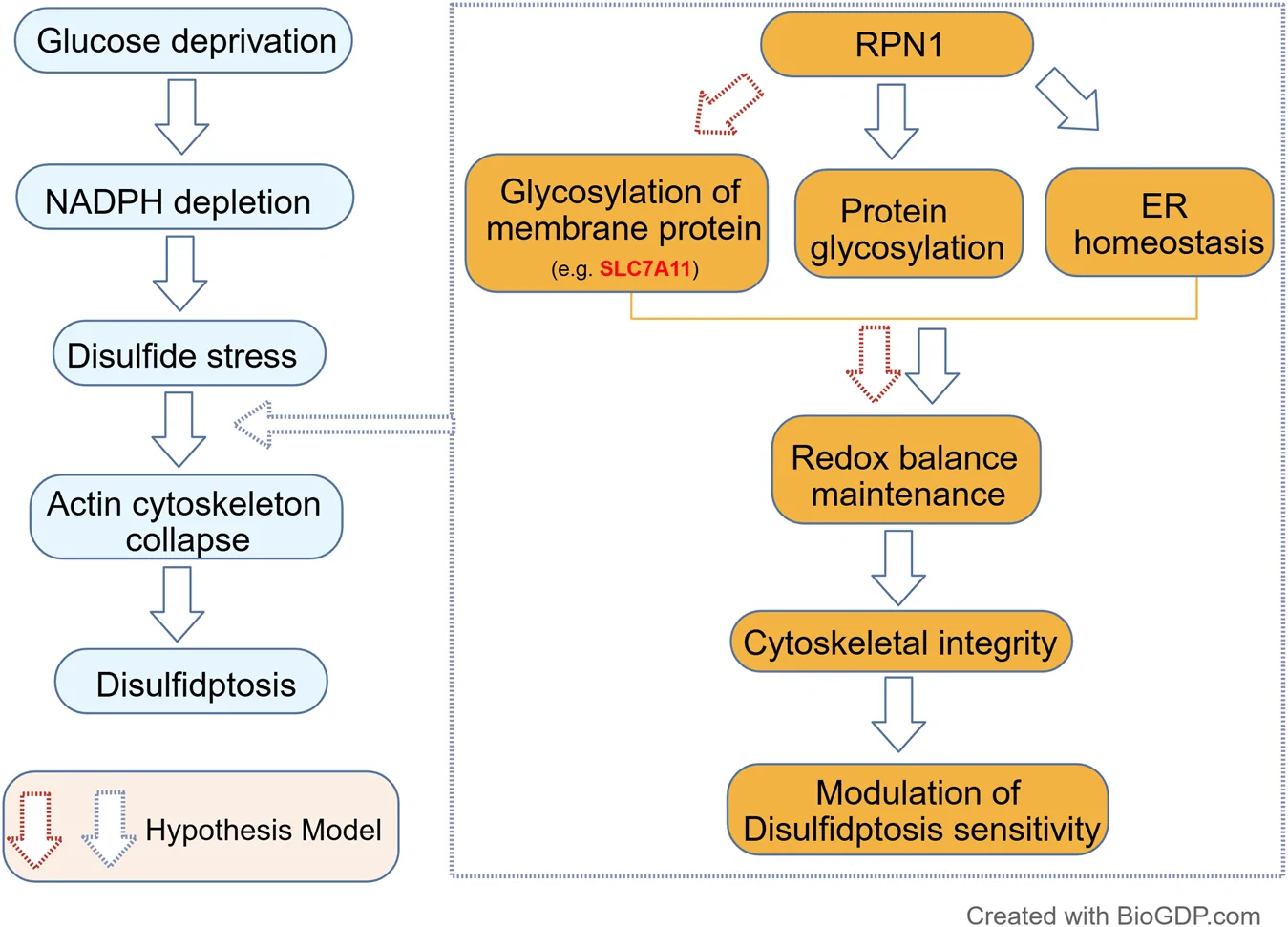
Proposed model linking RPN1 and disulfidptosis. The left panel depicts the canonical pathway of disulfidptosis triggered by glucose deprivation: glucose limitation leads to NADPH depletion, disulfide stress, actin cytoskeleton collapse, and ultimately cell death via disulfidptosis. The right panel illustrates our proposed model of how RPN1 may modulate disulfidptosis sensitivity. As a core OST subunit, RPN1 maintains ER homeostasis and redox balance through its control of protein glycosylation, including that of membrane transporters such as SLC7A11. This regulation is hypothesized to preserve cytoskeletal integrity (potentially via Rac1-WAVE signaling), thereby influencing the cellular response to disulfide stress and disulfidptosis. Dashed arrows represent hypothetical regulatory connections that require further experimental validation.

At present, RPN1 can only be interpreted as a correlative biomarker associated with disulfidptosis-related gene signatures rather than a biochemically confirmed functional regulator of this cell death pathway. Future studies are needed to determine whether RPN1 directly influences sensitivity to glucose deprivation, disulfide stress, or cytoskeletal collapse, and to identify the molecular substrates responsible for these effects (Figure 4).

### 4.3 RPN1 in disulfidptosis-related prognostic models

Despite insufficient causal mechanistic evidence between RPN1 and disulfidptosis execution, multiple retrospective cohort analyses integrated RPN1 into disulfidptosis risk scoring systems for prognostic stratification and immunotherapy response prediction (Liu et al., 2025a).

In glioma, a three-gene risk score (LRPPRC, RPN1, GYS1) developed from TCGA, CGGA, and GEO cohorts divides patients into high-risk subgroups with inferior survival and immunosuppressive microenvironments enriched in M2 macrophages and reduced CD8^+^ T infiltration (Wang et al., 2023a). Follow-up cellular assays further validated RPN1’s pro-proliferative and migratory functions in glioma cell lines (Niu et al., 2025).

In HCC, multiple independent LASSO-Cox regression models identified RPN1 as an independent poor prognostic factor within four-gene disulfidptosis signatures (LRPPRC, NCKAP1, RPN1, SLC7A11), which correlates with elevated immune checkpoint expression and inferior overall survival (Huang et al., 2023). Multi-omics data further linked RPN1 with tumor stemness, aberrant methylation, and sorafenib resistance in HCC (He et al., 2024).

A disulfidptosis risk model based on RPN1, SLC7A11, and GYS1 has been shown to reliably stratify prognosis in TCGA and ICGC cohorts as well as in clinical specimens, and it also predicts immunotherapy response, with a clear mechanistic link established between the hsa-miR-143-3p/LINC00511 axis and RPN1-mediated immune evasion (Xu et al., 2024). In bladder cancer, a three-gene risk model (NDUFA11, RPN1, SLC3A2) predicted overall survival and, when integrated with IMvigor210 immunotherapy cohort data, identified low-risk patients more likely to respond to PD-L1 inhibitors (Jiang et al., 2023).

Collective signature correlation data place RPN1 at the intersection of disulfide stress signatures and tumor immune phenotypes, providing preliminary translational value for prognostic stratification, though direct functional linkage between RPN1 and disulfidptosis remains unproven.

## 5 RPN1 and immunotherapy response

### 5.1 Correlation with immune checkpoint inhibitor efficacy

Emerging evidence suggests that RPN1 may be associated with the response to immune checkpoint inhibitors (ICIs), although its predictive value appears to vary across tumor types.

The fact that elevated RPN1 expression in glioma and esophageal cancer is correlated with poor response to PD-1/PD-L1 blockade (Lin et al., 2025) suggests a plausible connection to the well-documented relationship between RPN1 and an immunosuppressive tumor microenvironment rich in M2 macrophages and poor in CD8^+^ T cells. Since these immune features are themselves linked to limited responsiveness to checkpoint inhibition, this link is both logical and compelling.

The findings from bladder cancer research deliver contradictory correlative results: as several studies have now shown that disulfidptosis-related risk models incorporating RPN1 can reliably stratify patients according to immunotherapy outcomes (Jiang et al., 2023). Moreover, tumors with higher RPN1 expression in various cohorts display features of increased immunogenicity: higher tumor mutational burden (TMB) and greater immune signaling activity (Luo et al., 2025). Therefore, it is very reasonable to consider that the biological consequences of RPN1 expression depend critically on the tumor context.

Such tumor-type divergent effects are likely shaped by tissue-specific genomic backgrounds, immune cell composition and intrinsic metabolic states. At current stage, only correlative cohort evidence supports a statistical association between RPN1 abundance and ICI efficacy; prospective multi-center clinical cohorts and tumor-type specific mechanistic research are required before RPN1 can be applied as universal predictive biomarker for patient selection.

### 5.2 Experimental evidence for targeting RPN1 to enhance immunotherapy

Whether active intervention in RPN1 function improves immunotherapy is a central and important translational question, and the most convincing evidence so far comes from Wang et al., who thoroughly investigated the effects of RPN1 knockout on immune microenvironment remodeling and anti-PD-1 therapy enhancement in TNBC models (Wang M. et al., 2025). Specifically, shRNA-mediated RPN1 silencing in TNBC cell lines inhibited proliferation, colony formation, migration, and invasion. More importantly, RPN1 knockout dramatically reduced cell-surface PD-L1 without affecting total mRNA, thereby elegantly demonstrating that RPN1 regulates PD-L1 stability at the post-translational level (Wang M. et al., 2025).

Co-immunoprecipitation and glycosylation biochemical assays verified RPN1 participates in PD-L1 N-glycosylation as a core OST component (Wang M. et al., 2025). Therefore, RPN1 loss triggers PD-L1 deglycosylation, accelerates ubiquitin-mediated proteasomal degradation and weakens membrane PD-L1 immunosuppressive function (Wang M. et al., 2025).

In 4T1 syngeneic tumor-bearing mice, RPN1knockout reshapes the immune microenvironment: intratumoral M2 macrophages and regulatory T cells decrease significantly, while CD8^+^T cell infiltration and effector cytokines IFN-γ/Granzyme B increase, converting the immunosuppressive microenvironment to a “hot” tumor state (Wang M. et al., 2025).

More importantly, combining RPN1 knockout with anti-PD-1 antibody in tumor-bearing mice produced markedly superior tumor growth inhibition and survival prolongation compared with either intervention alone, with complete remission in some animals. Histological staining confirmed elevated intratumoral CD8^+^ T cell infiltration and reduced membrane PD-L1 expression in the combination group (Wang M. et al., 2025).

The YY1/RPN1/YBX1 regulatory axis concurrently elevates RPN1 expression and stabilizes PD-L1, forming an integrated immune escape cascade (Wang M. et al., 2025). Preclinical TNBC model data solidify the rationality of RPN1 targeted intervention to relieve PD-L1-mediated immunosuppression and boost checkpoint blockade efficacy.

### 5.3 Interactions with chemotherapy and radiotherapy

High RPN1 expression correlates with temozolomide resistance in glioma, mediated by glycosylation stabilization of drug efflux transporters ABCB1/ABCC1 and modified DNA damage response protein glycan profiles, reducing intracellular chemotherapeutic accumulation (Rezaei et al., 2024). Thus, RPN1 may acts as an negative regulator of chemotherapy sensitivity in the highly heterogeneous, treatment-resistant tumors such as GBM (Li M.-Y. et al., 2025; Mahomoodally et al., 2025; Li et al., 2026).

In high-grade serous ovarian carcinoma, RPN1 overexpression correlates with inferior platinum-taxane response (Luo et al., 2025). Although direct RPN1–chemotherapy response data are sparse, glycosylation modifications are known to regulate platinum drug targets and apoptotic pathway molecules (Liu Z.-P. et al., 2025); RPN1 likely influences ovarian cancer cell responses to platinum–taxane regimens through similar mechanisms. Whether RPN1 modulates redox-related proteins such as SLC7A11 or GPX4 to affect chemotherapy-induced oxidative stress tolerance warrants investigation (Zhang et al., 2024; Mahomoodally et al., 2025; Wang J. et al., 2025).

RPN1 may serve as a very clear and elegant bridge between chemoradiotherapy and immunotherapy synergy: radiotherapy releases neoantigens and activates the STING pathway, thereby promoting T cell infiltration (Ji et al., 2023), but concomitant RPN1- mediated PD-L1 stabilization neutralizes this immune-activating effect. Therefore, inhibiting RPN1 weakens PD-L1 membrane stability, lifts T cell suppression, and thus amplifies the radiotherapy-induced abscopal effect (Luo et al., 2025). In glioma, where combined TMZ, radiotherapy, and ICI therapies are limited by severe immunosuppression (Rezk et al., 2025), RPN1 targeting offers a way to remodel the tumor microenvironment from cold to hot, which should improve triple-therapy response rates.

The link to disulfidptosis opens another combination avenue. Under glucose deprivation, SLC7A11-high tumor cells undergo disulfidptosis; as a DRG risk model component, RPN1 may modulate sensitivity to redox imbalance by glycosylating cytoskeletal proteins such as ACTB and VIM (Luo et al., 2025). RPN1 expression could thus serve as a stratification biomarker for metabolic intervention plus immunotherapy combinations (Wang J. et al., 2025; Wu et al., 2025; Zhang et al., 2026).

Since RPN1 affects chemotherapy sensitivity and functions as a multi-dimensional regulatory node in radiotherapy–immunotherapy crosstalk, targeting RPN1 provides a rational approach to break single-treatment limitations, thereby inducing synergy via enhanced DNA damage, relief of immunosuppression, and induction of novel cell death. Thus, systematic studies in additional cancer types are necessary for proper clinical translation.

## 6 Therapeutic strategies and translational prospects

### 6.1 Challenges and paths for direct RPN1 targeting

Despite growing interest in RPN1 as a therapeutic target, several challenges currently limit its clinical translation. RPN1 is an endoplasmic reticulum (ER)-resident membrane protein that functions within the multi-subunit oligosaccharyltransferase complex. Unlike many enzymes, it lacks a well-defined catalytic pocket that can be readily targeted by conventional small molecules (Liu et al., 2025a). In addition, because RPN1 participates in fundamental cellular glycosylation processes, broad inhibition may result in undesired toxicity in normal tissues.

RNA-based approaches offer a clear and powerful strategy for selective RPN1 suppression, and evidence from TNBC models shows that siRNA- or shRNA-mediated RPN1 knockdown decreases PD-L1 glycosylation and inhibits tumor progression (Wang M. et al., 2025). However, the major hurdle remaining is efficient delivery. Although lipid nanoparticle systems have been successfully used in several RNA therapeutics, reliable, tumor-specific delivery to cancers such as glioma and sarcoma is still a significant challenge (Yan et al., 2025).

Targeted protein degradation technologies have also attracted interest. Proteolysis-targeting chimeras (PROTACs) have successfully degraded numerous intracellular proteins by recruiting E3 ubiquitin ligases to target proteins (Békés et al., 2022). In principle, similar approaches could be explored for RPN1. However, the feasibility of degrading an ER-resident membrane protein using current PROTAC platforms remains uncertain. To date, no RPN1-targeting PROTAC has been reported, while CAP-TACs can recruit proteins to RPN1 for ubiquitination-independent degradation, classic E3-dependent PROTACs targeting RPN1 itself remain undisclosed (Song et al., 2026). And substantial technical challenges related to intracellular accessibility, membrane localization, and degradation efficiency remain unresolved (Li et al., 2023; Jia et al., 2024; Wang and Liao, 2024; Zhu B.-R. et al., 2025).

Alternative degradation platforms, including lysosome-targeting chimeras (LYTACs) and autophagy-targeting chimeras (AUTACs), may offer additional opportunities for membrane protein targeting. Nevertheless, these approaches remain largely experimental in the context of RPN1.

Because of the present limitations, indirect targeting strategies are currently more practical than direct RPN1 inhibition, and therefore interfering with downstream pathways regulated by RPN1, such as PD-L1 glycosylation, ER stress signaling, or immune suppressive programs, represents a more immediately translatable therapeutic approach. Moreover, ongoing progress in RNA delivery, targeted protein degradation, and glycosylation-directed drug development is likely to broaden the therapeutic options available for RPN1-associated tumors.

Direct pan-RPN1 inhibition disrupts physiological glycan modification across normal immune, epithelial and stromal cells, leading to broad side effects and narrow therapeutic windows. Future translational research needs to prioritize tumor-specific delivery carriers to restrict RPN1 suppression exclusively within malignant lesions. Novel micro-robot delivery systems provide theoretical support for tumor-specific nucleic acid and protein drug targeted transportation, which can be referenced for optimizing RPN1 silencing vector design (Wang and Liao, 2024).

### 6.2 Biomarker development

Cumulative retrospective cohort correlative data support RPN1’s multi-dimensional biomarker value for prognosis, ICI response prediction and combinatorial therapy guidance across HCC, GBM, sarcoma and bladder cancer (Jiang et al., 2023). In bladder cancer, RPN1 is part of a well-validated three-gene risk score (NDUFA11, RPN1, SLC3A2) that reliably stratifies patients by risk and accurately predicts overall survival, as cross-validated by the Human Protein Atlas (Jiang et al., 2023). Similarly, in gastric cancer, RPN1 combines with SLC7A11 and NCKAP1 in prognostic systems with broad applicability across gastrointestinal tumors (Yan et al., 2024; Chen Y. et al., 2025).

RPN1 expression level predicts divergent ICI response across tumor subtypes: high RPN1 levels correlates with immunosuppressive microenvironments and anti-PD1 resistance in glioma/esophageal cancer, while correlative data suggest favorable checkpoint inhibitor response in bladder cancer; systematic subtype-specific mechanistic research is required to resolve this discrepancy, with RPN1-mediated PD-L1 glycosylation serving as a core underlying explanatory pathway (Jiang et al., 2023; Chen Y. et al., 2025).

RPN1 can reasonably be considered a guide for combination therapy decisions because it is involved in DNA repair, ER stress, and redox homeostasis, hence RPN1 levels may serve as a biomarker for sensitivity to chemotherapy, radiotherapy, or disulfidptosis inducers (Chen Y. et al., 2025). Importantly, in gastric and liver cancer, the RPN1–SLC7A11 co-expression pattern identifies patient subgroups that are more responsive to oxidative stress-targeted interventions, allowing for truly personalized combination strategies (Yan et al., 2024; Chen Y. et al., 2025).

Liquid biopsy applications are advancing nicely, with analysis of RPN1 expression or transcripts in PBMCs, ctDNA, or exosomes offering a clear path for non-invasive, dynamic monitoring. More importantly, glycosylation-related gene expression in PBMCs serves as a robust biomarker for the tumor microenvironment state (Mohapatra, 2023; Ho et al., 2024; Ravilla et al., 2024; Gope et al., 2025b; Pandey and Yadav, 2025), and RPN1, being a core OST component, thus acts as a real-time window into tumor burden, immune status, and treatment response. Though direct ctDNA/exosomal RPN1 detection assays remain unreported, integrating RPN1 with PD-L1/SLC7A11 multi-analyte liquid biopsy panels is technically feasible via NGS and digital PCR for early recurrence surveillance and immunotherapy dynamic monitoring (Ho et al., 2024; Ravilla et al., 2024; Gope et al., 2025b).

Large-scale prospective multi-center clinical cohorts and standardized IHC/digital PCR detection cut-off values are required before RPN1 can be translated into routine clinical predictive biomarkers, and liquid biopsy detection systems require further technical optimization. General background of traditional and modern tumor therapeutic systems can be supplemented by research on food-medicine homology anti-tumor strategies (Li M.-Y. et al., 2025); ferulic acid-mediated tumor proliferation and cell cycle suppression research provides auxiliary reference for small molecule adjuvant therapy discussion (Liu Z.-P. et al., 2025).

### 6.3 Future directions for RPN1-Targeted combination immunotherapy

As a core modulator of PD-L1 glycosylation and immunosuppressive TAM polarization, RPN1 represents an attractive combinatorial target alongside existing immune checkpoint blockade. Consequently, future strategies may combine RPN1 inhibition with existing ICI therapies and other immunomodulators to overcome the limitations of monotherapy and convert cold tumors into hot ones. Single-agent PD-1/PD-L1 blockade has limited efficacy in many solid tumors precisely because of the persistent immunosuppressive microenvironment and the aberrant stability of PD-L1 (Gope et al., 2025a). More importantly, RPN1-mediated N-glycosylation promotes PD-L1 membrane localization and function, so targeting RPN1 should weaken PD-L1-mediated immunosuppression and thus sensitize tumors to checkpoint blockade. Preclinical TNBC data confirm RPN1 inhibition downregulates membrane PD-L1 and converts immune cold tumors to treatment-responsive phenotypes, generating synergistic effects with checkpoint inhibitors (Lu, 2025).

RPN1-targeted strategies lend themselves very naturally to combination with other immunomodulators because emerging checkpoints such as LAG-3, TIM-3, and TIGIT are frequently co-expressed with PD-1 on exhausted T cells, and together they form multi - layered immunosuppressive networks (Yue, 2024). Since RPN1 can modulate these checkpoints via ER stress and inflammatory signaling pathways, multi-target checkpoint blockade combined with RPN1 inhibition might therefore achieve more complete immune activation. Moreover, RPN1’s role in TAM polarization makes it an excellent candidate for combination with CSF-1R inhibitors or CD40 agonists to reverse myeloid-mediated immunosuppression (Lu, 2025).

Synergy between RPN1 inhibition and disulfidptosis induction delivers dual metabolic-immunological anti-tumor benefits: glucose deprivation triggers tumor-specific disulfide stress death while RPN1 silencing relieves PD-L1-mediated immune suppression, releasing tumor neoantigens to amplify ICI response rates, fitting the dual immunotherapy plus metabolic sensitizer combinatorial paradigm (Wang Y. et al., 2025).

Clinical translation of this approach will necessarily involve multomics-based patient stratification, selecting patients with high RPN1 expression, active PD-L1 glycosylation, or positive disulfidptosis gene signatures (He et al., 2024; Li Q. et al., 2025). At the same time, the development of RPN1-targeted small molecules, siRNA delivery systems, or PROTAC degraders, along with the use of nanotechnology for optimal delivery, has been systematically explored (Zhong et al., 2024; Boti et al., 2025; Jan et al., 2026; Shaik et al., 2026). Hence, integrating glycosylation regulation, immune checkpoint blockade and metabolic cell death induction, RPN1-targeted combinatorial therapy opens novel pipelines for overcoming refractory tumor immunotherapy bottlenecks.

## 7 Conclusion and perspectives

### 7.1 RPN1 as a multi-modal tumor driver

Accumulating transcriptomic, proteomic, clinical cohort and partial *in vitro*/*in vivo* functional data consistently identify elevated RPN1 expression in glioma, HCC, sarcoma and TNBC, correlated with malignant clinicopathological phenotypes and inferior survival (Lin et al., 2025).

Recent studies have considerably broadened the functional profile of RPN1, showing that in addition to its well-known role in protein homeostasis, it is involved in immune evasion, remodeling of the tumor microenvironment, ER stress adaptation, and genomic instability-related phenotypes. More importantly, RPN1 regulates PD-L1 glycosylation, thereby providing a clear and elegant mechanism by which glycosylation-dependent processes can influence anti-tumor immunity (Wang M. et al., 2025). Complementing this, there is now more evidence linking RPN1 expression to macrophage polarization, CD8^+^ T-cell exclusion, and immunotherapy response, suggesting that RPN1 plays a general role in shaping tumor-immune interactions across many cancer types (Lin et al., 2025).

Multiple disulfidptosis prognostic signatures integrate RPN1 as a core gene component, though direct experimental evidence confirming its causal regulatory role in disulfide stress cell death remains absent (Wang et al., 2023b; Qin et al., 2024; Xu et al., 2024). Despite unresolved mechanistic gaps, existing tiered evidence (biochemical functional validation + multi-omics correlative analysis) supports RPN1 as a promising dual candidate for prognostic biomarker and targeted therapeutic intervention in precision oncology.

### 7.2 Outstanding questions and future perspectives

Although much has been learned about the biological and clinical importance of RPN1, it is undeniable that several major questions remain to be answered.

- The question arises: is RPN1 a functional regulator of disulfidptosis or merely a biomarker?
- The most striking and interesting finding to date is the consistent appearance of RPN1 in disulfidptosis-related gene signatures across many cancer types, but it must be noted that present evidence is mainly based on bioinformatic analyses and prognostic modeling, so the direct role of RPN1 in disulfide stress, cytoskeletal collapse, or cellular vulnerability to glucose deprivation remains unproven. Therefore, establishing a mechanistic link between RPN1 and disulfidptosis requires rigorous functional studies employing genetic and pharmacological methods.
- Which RPN1-dependent glycoproteins are most relevant to tumor immunity? PD-L1 is currently the best-characterized immune-related substrate associated with RPN1 activity. Nevertheless, RPN1 participates in the glycosylation of a broad range of membrane and secreted proteins. It remains unclear whether additional immune regulatory molecules-including cytokine receptors, adhesion molecules, antigen-presentation machinery, or alternative immune checkpoints-are similarly affected by RPN1-dependent glycosylation. Systematic glycoproteomic analyses may help identify the most biologically relevant downstream effectors.
- What is the reason for the different association of RPN1 with immunotherapy response in different tumor types? Current literature indicates that elevated RPN1 expression is linked to poor response to immune checkpoint inhibitors in some cancers, but opposite results have been reported in other cancer types, and thus the biological basis for this discrepancy remains unclear. Likely explanations include differences in tissue origin, genomic background, immune cell composition, metabolic state, and co-regulated signaling pathways, all of which can give rise to context-dependent effects. Therefore, comparative analyses across tumor types are necessary to determine whether RPN1 acts as a driver, a modifier, or merely a marker of immune responsiveness.
- Is it possible to therapeutically target RPN1 with adequate selectivity?
- Although RPN1 is an excellent candidate target, its universal role in protein glycosylation naturally raises important concerns about toxicity and the therapeutic window, hence the key question to address is whether cancer cells rely more heavily on RPN1 than normal tissues. Therefore, future strategies are likely to focus on tumor-selective delivery systems, context-specific vulnerabilities, or downstream pathways that can be targeted without causing widespread systemic effects.
- How should RPN1-based biomarkers be translated into clinical practice? Numerous retrospective studies have demonstrated associations between RPN1 expression and prognosis, immune infiltration, or treatment response. However, prospective clinical validation remains limited. Standardized detection methods, clinically relevant cut-off values, and validation in large multicenter cohorts will be necessary before RPN1 can be incorporated into routine clinical decision-making. In addition, the potential value of RPN1 in liquid biopsy platforms, including circulating tumor DNA, exosomal RNA, and peripheral blood-based assays, remains largely unexplored.

Since answering these questions will enhance our understanding of RPN1 biology and at the same time help clarify RPN1’s role in precision oncology, and given that current knowledge about tumor glycosylation, immune regulation, and metabolic vulnerabilities is growing rapidly, RPN1 is sure to remain a major focus of future translational research.

Despite substantial progress in understanding the biological and clinical relevance of RPN1, several important questions remain unresolved.

### 7.3 Future research priorities

Future investigations should focus on three major directions. First, defining the direct glycoprotein substrates regulated by RPN1 through quantitative glycoproteomics will be critical for understanding how RPN1 remodels tumor immunity. Second, mechanistic studies are needed to determine whether RPN1 actively participates in disulfidptosis regulation or merely serves as a surrogate biomarker of disulfidptosis-related transcriptional programs. Third, translational efforts should evaluate RPN1-targeted therapeutic strategies, including RNA-based therapeutics, targeted protein degradation technologies, and combination regimens with immune checkpoint blockade.

From the available studies it is possible to determine whether RPN1 can legitimately evolve from a prognostic biomarker into a clinically useful therapeutic target.
